# Role of the Eye in Transmitting Human Coronavirus: What We Know and What We Do Not Know

**DOI:** 10.3389/fpubh.2020.00155

**Published:** 2020-04-24

**Authors:** Chuan-bin Sun, Yue-ye Wang, Geng-hao Liu, Zhe Liu

**Affiliations:** ^1^Eye Center, Second Affiliated Hospital of Zhejiang University School of Medicine, Hangzhou, China; ^2^Department of Ophthalmology, Zhejiang Provincial People's Hospital, People's Hospital of Hangzhou Medical College, Hangzhou, China

**Keywords:** Coronavirus, 2019-nCoV, SARS-CoV-2, transmission, infection, conjunctiva, conjunctivitis, eye

## Abstract

The outbreak of the current 2019 novel coronavirus (2019-nCoV, now named SARS-CoV-2) infection has become a worldwide health threat. Currently, more information is needed so as to further understand the transmission and clinical characteristics of 2019-nCoV infection and the infection control procedures required. Recently, the role of the eye in transmitting 2019-nCoV has been intensively discussed. Previous investigations of other highly infectious human CoVs, that is, severe acute respiratory syndrome coronavirus (SARS-CoV) and the Middle East respiratory syndrome coronavirus (MERS-CoV), may provide useful information. In this review, we describe the genomics and morphology of human CoVs, the epidemiology, systemic and ophthalmic manifestations, and mechanisms of human CoV infection, and recommendations for infection control procedures. The role of the eye in the transmission of 2019-nCoV is discussed in detail. Although the conjunctiva is directly exposed to extraocular pathogens, and the mucosa of the ocular surface and upper respiratory tract are connected by the nasolacrimal duct and share the same entry receptors for some respiratory viruses, the eye is rarely involved in human CoV infection, conjunctivitis is quite rare in patients with 2019-nCoV infection, and the CoV RNA positive rate by RT-PCR test in tears and conjunctival secretions from patients with 2019-nCoV and SARS-CoV infection is also extremely low. This suggests that the eye is neither a preferred organ of human CoV infection nor a preferred gateway of entry for human CoVs for infecting the respiratory tract. However, pathogens that the ocular surface is exposed to might be transported to nasal and nasopharyngeal mucosa by constant tear rinsing through the lacrimal duct system and then cause respiratory tract infection. Considering that close doctor-patient contact is quite common in ophthalmic practice and is apt to transmit human CoVs by droplets and fomites, strict hand hygiene and proper personal protection are highly recommended for health care workers to avoid hospital-related viral transmission during ophthalmic practice.

## Introduction

Coronavirus (CoV) is an enveloped single-stranded positive-sense RNA virus that typically causes respiratory and enteric infections affecting both human and wild animals (1–3). Since first being identified in the 1960s, Human CoVs were considered relatively benign and usually caused mild upper respiratory tract infections (the common cold) until the emergence of the severe acute respiratory syndrome coronavirus (SARS-CoV) in 2002 and, later, the Middle East respiratory syndrome coronavirus (MERS-CoV) in 2012 (4). The latter two CoVs can result in severe lower respiratory tract infection, rapidly proceeding to pneumonia, and have caused thousands of cases of infection and hundreds of deaths in about 30 countries, respectively (2, 4). In December 2019, another outbreak of highly infectious pneumonia caused by a novel coronavirus (2019-nCoV, now named as SARS-CoV-2) emerged in Wuhan, China, and soon became a major global health threat (2, 3).

Currently, more detailed information about the transmission of 2019-nCoV is urgently needed to prevent its pandemic spread. Human CoVs mostly spread through respiratory droplets expelled by infected individuals and direct contact with virus-contaminated fomites (4). Anatomically, the conjunctiva of the eye is easily exposed to infectious droplets and fomites during close contact with infected individuals and contaminated hands. Some respiratory viruses such as human adenovirus (species D) and avian influenza virus (H7) frequently cause highly infectious conjunctivitis or keratoconjunctivitis. Hence, conjunctiva is postulated to be an important portal of entry for respiratory viruses, while tear and conjunctival secretions may contain virus and spread viral infection (4, 5).

However, the role of the eye in the transmission of human CoVs is still under discussion, as considerable controversy exists. This review presents the genomics and morphology of human CoVs, the epidemiology, systemic and ophthalmic manifestations, and mechanisms of human CoV infection, and the role of the eye in the transmission of human CoVs. Infection control procedures and personal protective equipment against human CoV transmission in ophthalmic practice are also reviewed.

## Genomics

CoVs have an enveloped single positive-strand RNA genome with a 5′-terminal cap structure and a poly(A) sequence at the 3′ end. CoV genome is approximately 30 kb (27~32 kb) long and is the largest RNA genome known so far (1, 4, 6). CoVs belong to the family *Coronaviridae* and the order *Nidovirales* and are classified into four genera: α-CoV, β-CoV, γ-CoV, and δ-CoV (1, 6, 7).

Until now, a total of seven human CoVs have been identified, namely HCoV-229E, HCoV-NL63, HCoV-OC43, HCoV-HKU1, SARS-CoV, MERS-CoV, and, recently, 2019-nCoV (1–3, 6–8). The former two human CoVs belong to the genus α-CoV, and the latter five human CoVs belong to the genus β-CoV. Three recently identified human CoVs, that is, SARS-CoV, MERS-CoV, and 2019-nCoV, have been recognized as zoonotic viruses, which transmit between animals and human. Recent studies revealed that SARS-CoV was transmitted from civet cats to humans, MERS-CoV from dromedary camel, and 2019-nCoV (probably) from pangolin (1, 2, 6–9). Recent investigations indicated that bats were most probably the natural reservoir of SARS-CoV, MERS-CoV, and 2019-nCoV (1, 6, 9–11). Genome sequence analysis revealed that 2019-nCoV was distinct from SARS-CoV (about 79% identity) and MERS-CoV (about 50% identity) yet more closely related to SARS-like-CoVs (about 88% identity) in bats (10, 11).

## Morphology

CoV particles have a spherical or elliptical shape with a diameter of about 100 nm (50~200 nm). They carry three major structural proteins in the envelope and contain a helical nucleocapsid formed by the viral genomic RNA and the viral nucleoprotein. The viral spike protein has receptor-binding and fusogenic functions and is essential for initiation of CoV infection (1, 8, 12–14). Further three-dimensional structure analyses suggest that the spike protein is composed of two subunits: S1, which mediates SARS-CoV binding to receptors on host cell membranes, and S2, which triggers the membrane fusion between the virus and host cells (11, 13).

## Epidemiology

Four human CoVs, that is, HCoV-229E, HCoV-NL63, HCoV-OC43, and HCoV- HKU1, are usually low in infectiousness and primarily infect the upper respiratory tract, causing mild respiratory symptoms (the common cold), whereas the other three human CoVs, SARS-CoV, MERS-CoV, and 2019-nCoV, are zoonotic and highly infectious and predominantly cause severe lower respiratory tract infection which can rapidly proceed to pneumonia (1–3, 8, 15, 16). The outbreak of SARS in 2002 in China resulted in 8,098 cases and 774 deaths (case-fatality rate, 9.6%) in 37 countries, and the outbreak of MERS in 2012 in Middle East Countries led to 2,494 cases and 858 deaths (case-fatality rate, 34%) in 27 countries (2). As of February 24, 2020, 2019-nCoV has caused 77,262 cases and 2,595 deaths in China, and 2,069 cases and 23 deaths in 29 other countries (total case-fatality rate, 3.3%) (15–17). Hence, although 2019-nCoV can cause a severe respiratory disease like SARS and MERS, it appears to be less pathogenic than SARS-CoV and much less so than MERS-CoV. However, the number of 2019-nCoV infected patients in the first two months was nearly 10 times that of SARS patients in total, which indicated that 2019-nCoV is more transmissible than SARS-CoV and MERS-CoV (16).

Human CoVs primarily spread by virus-containing droplets or aerosols expelled by infected individuals when patients cough, talk loudly, or sneeze. Direct contact with virus-contaminated fomites is also a route of human CoV transmission (4, 8, 18–20). Recently, SARS-CoV and 2019-nCoV have also been detected in stool and urine samples from patients by RT-PCR assay and have been isolated from the mucous membranes of gastrointestinal tract in a few cases (9, 16, 21). Hence, fecal-oral route may also be a route of transmission for SARS-CoV and 2019-nCoV.

## Clinical Manifestations

The clinical features of coronavirus disease 2019 (CoVID-19) are similar to those of SARS and MERS. Most patients present with fever, dry cough, dyspnoea, and bilateral ground-glass opacities on chest CT scans (2, 3, 22–24). However, CoVID-19 rarely results in notable symptoms of upper respiratory tract infection (e.g., rhinorrhea, sneezing, or sore throat), which are commonly manifested in SARS and MERS. Some CoVID-19 patients even manifest no apparent respiratory symptoms at onset, which never occurred in SARS and MERS (24–26). Mathematical models have revealed that the 2019-nCoV virus may replicate very slowly in the first days after infection and that it could be below detection levels during the first four days post infection (26).

Recent investigations have also revealed that CoVID-19 occasionally manifests as enteric infection symptoms such as diarrhea, whereas about 20~25% of patients with MERS or SARS had diarrhea (25). Moreover, more than 80% of CoVID-19 has manifested as mild or moderate pneumonia, and the severe CoVID-19 has mostly occurred in the patients of over 60 years old, usually accompanied by at least one underlying disorder, for example, cardiovascular disorders, diabetes, chronic obstructive pulmonary disease, and hypertension (24).

## Ophthalmic Manifestations

The eye is rarely involved in human CoV infection. Until now, conjunctivitis has been reported in only five cases with 2019-nCoV infection, and in four cases with HCoV-NL63 infection, whereas no conjunctivitis or other ocular complications have been reported in patients with SARS-CoV and MERS-CoV infection (4, 27–31). Recently, human CoV RNA in tears and conjunctival scraping samples were tested by reverse transcription-polymerase chain reaction (RT-PCR) assay in patients with SARS and CoVID-19, yet the positive rate of the RT-PCR test was extremely low (4, 30–37).

Loon and colleagues detected SARS-CoV in tear samples from 36 consecutive SARS suspects (eight patients were laboratory-confirmed later) by RT-PCR (32). SARS-CoV was positive only in three of the eight SARS cases. Three patients whose tears were SARS-CoV positive were sampled in the early phase of their illness (on Days 3, 4, and 9 after onset of fever, respectively), whereas the other five SARS cases, whose tears were SARS-CoV negative, were sampled in the later phase (mean 19.4 days) of their illness (32). Nearly at the same time, Chan and colleagues reported their negative results of SARS-CoV testing in tear and conjunctival scraping samples from 20 probable SARS patients (17 patients were laboratory-confirmed later) by RT-PCR and virus culture (33). Among 17 confirmed SARS patients, 6, 8, and 3 cases were recruited during the first, second, and third week of their diseases, respectively. SARS-CoV RNA was not detected by RT-PCR and SARS-CoV was not isolated in virus culture in any of the tear and conjunctival scraping samples (33). Leong and colleagues tested for SARS-CoV in 126 conjunctival specimens from 64 SARS patients in the convalescent phase by RT-PCR but did not detect SARS-CoV in any of the patients' conjunctival samples (22).

On January 22, 2020, a Chinese respiratory specialist who visited Wuhan as a member of the national expert panel on pneumonia claimed that he was infected by 2019-nCoV despite being fully gowned with a protective suit and N95 respirator (34). His first clinical manifestation was unilateral conjunctivitis, followed by fever and catarrhal symptoms 2 or 3 h later. He postulated that 2019-nCoV probably first infected the conjunctiva, then spread and cause viral pneumonia (34). Soon after his report, health care personnel in China were urged to use eye protection when they were in close contact with CoVID-19 patients or suspected patients. However, Zhou and colleagues, in a preprint posted at medRxiv, reported that conjunctivitis was identified only in one patient out of 63 CoVID-19 cases and 4 suspected CoVID-19 cases (27). Conjunctivitis was also the first symptom of 2019-nCoV infection in this patient. However, 2019-nCoV RNA tested by RT-PCR was positive and probably positive in conjunctival swab samples from only one and two CoVID-19 cases without conjunctivitis, respectively. None of the above three patients had ocular symptoms. 2019-nCoV RNA was not detected in conjunctival swab samples from the CoVID-9 patient complicated by conjunctivitis, who was an anesthesiologist. Her ocular symptoms occurred soon after performing tracheal intubation for a patient who was confirmed as having CoVID-19 later, and this was followed by fever and cough. Unfortunately, the personal protections used by this anesthesiologist during the tracheal intubation procedures were only a surgical mask, cap, and gloves, without a gown, face shield or goggles. Her five colleagues were also infected by the same patient, yet none of them exhibited any ocular complications (27).

More recently, two investigative groups from China simultaneously reported conjunctivitis and 2019-nCoV RNA-positive tests in conjunctival swab samples from CoVID-19 patients (28, 31). Zhang and colleagues, in a preprint posted at medRxiv, reported conjunctivitis in two patients out of 72 laboratory-confirmed CoVID-19 cases; however, 2019-nCoV was detected in conjunctival swab samples by RT-PCR in only one patient who was a nurse working in the Emergency Department (28). This patient presented with excessive tearing and redness in both eyes, which were typical ocular manifestations of viral conjunctivitis, accompanied by a moderate fever of 38.2°C that occurred 1 day earlier. 2019-nCoV RT-PCR tests for the conjunctival and oropharyngeal swabs sampled 2 days after the onset of fever was positive, but for those sampled 9, 18, and 20 days after the onset of fever were all negative (28). Xia and colleagues reported unilateral conjunctivitis in one patient out of 30 confirmed CoVID-19 cases; conjunctival swabs sampled from this patient 3 and 5 days after the onset of CoVID-19 were both positive for 2019-nCoV by RT-PCR, whereas 58 conjunctival swab samples from the other 29 CoVID-19 patients were all negative for 2019-nCoV (31). However, 2019-nCoV was not isolated and cultured in the conjunctival swab samples from the CoVID-19 patient with conjunctivitis. In contrast, 55 of the 60 sputum samples from 30 CoVID-19 cases showed positive PCR results for 2019-nCoV (31).

Although tears have been reported by the World Health Organization in 2003 to be one of the body fluids that might contain SARS-CoV, the infectivity and clinical importance is not yet understood (35). Recent investigations have revealed that highly infectious human CoVs (mainly SARS-CoV and 2019-nCoV) are rarely detected by RT-PCR and never isolated by virus culture in tears and conjunctival secretions from SARS and CoVID-19 patients (27–34, 36). Hence, it is hard to assess the infectivity of tears and conjunctival secretions and their roles in virus transmission.

The extremely low positive rate of human CoV RNA test by RT-PCR in tears and conjunctival secretions from patients with SARS and CoVID-19 may have several interpretations. Firstly, the sensitivity of RT-PCR testing still needs to be improved. Previous reports on the sensitivity of RT-PCR in excretions reported a range from 50% to 60% (33, 37). In current clinical practice, some suspected 2019-nCoV cases often had 2~3 repeated tests of nasopharyngeal swabs before the positive results were obtained (28). The need remains for a highly sensitive and specific PCR test to diagnose human CoV infections. Secondly, the samples were not collected at the right time. Recent evidence indicated that human CoV RNA-positive cases were all sampled in the early part of the disease course, whereas human CoV RNA-negative cases sampled in the later or convalescent phase of their illness (33). de Wit and colleagues demonstrated that, based on their rhesus macaque model study, MERS-CoV RNA could be detected in the conjunctiva only within 6 days post infection (38). Hence, it is reasonable to postulate that human CoV may present in tears only for a short period during the early phase of the disease. Thirdly, the contribution of antimicrobial agents, including lactoferrin and secretory IgA, in tears and constant tear rinsing, which continuously eliminates the virus on the ocular surface into the nasal cavity through the nasolacrimal duct (37, 39, 40), should be considered. Lactoferrin can inhibit the binding of SARS-CoV to its entry receptor, angiotensin-converting enzyme 2 (ACE2), by preventing the adhesion of SARS-CoV to its attachment receptor, heparan sulfate proteoglycans (HSPGs) (41). Secretory IgA is another important antimicrobial agent in tears that helps to kill both bacteria and viruses. The host immune system can be activated and result in a significant increase in lactoferrin and secretory IgA levels in tears and circulating IgM level in plasm on the 3rd to 5th day and circulating IgG level in plasm on the 10th to 15th day after CoV infection or inoculation (39, 41), which may contribute to why CoV RNA presents only in the early phase of the disease. Fourthly, the collection technique may not appropriate. The World Health Organization highly recommends the use of only synthetic fiber swabs with plastic shafts rather than calcium alginate swabs or swabs with wooden shafts for specimen sampling, as the latter two types of swabs may contain substances that inactivate some viruses and inhibit PCR testing (40). Topical anesthesia is also not recommended for tear and conjunctival scraping sampling, for a topical anesthetic agent maybe also have a negative influence on the viability of viruses (40). Moreover, the volume of tears collected when sampling may also have some influence on the positivity of the RT-PCR test.

## Mechanisms of Human CoV Transmission

Anatomically, the mucosa of the ocular surface (i.e., conjunctival and corneal epithelia) and the upper respiratory tract are connected by the nasolacrimal duct (4). When dropped into the eye, liquid is partially absorbed by the cornea and conjunctiva but mostly drained into the nasal cavity through the nasolacrimal duct and then transported toward the lower part of the respiratory tract, including the nasopharynx and trachea, or swallowed into the gastrointestinal tract (37). This allows pathogens to which the eye is exposed to be transported to respiratory and gastrointestinal tract mucosa. Moreover, previous investigations have revealed that the mucosa of the ocular surface and respiratory tract share the same receptors for some respiratory viruses (4, 42–44). ACE2, the entry receptor of SARS-CoV, HCoV-NL63, and 2019-nCoV, is highly expressed on human lung alveolar epithelial cells, enterocytes of the small intestine, and the proximal tubular cells of the kidney (4, 42). Positive expression of ACE2 was also detected in human conjunctival and corneal epithelial cells; however, ACE2 expression in human ocular surface is much lower than in human lung and kidney tissues (43). The binding capability of ACE2 protein on conjunctival epithelial cells to SARS-CoV spike protein is much lower than that on Vero E6 cells and that in lung tissues (44).

The efficacy of virus entry into host cells depends on three points: the invasiveness of the virus, viral receptors on host cell membrane, and the immune conditions of the host. The virus binding to host cell membrane by its receptors is the first and key step for viral invasion. ACE2, a metallopeptidase, also the entry receptor of SARS-CoV, HCoV-NL63, and 2019-nCoV, is responsible for binding to spike protein on the SARS-CoV and HCoV-NL63 surface and mediating SARS-CoV and HCoV-NL63 entry into host cells (4, 11, 42–45), while MERS-CoV and most α-CoVs have been identified to utilize dipeptidyl peptidase 4 and aminopeptidase N as an entry receptor of their host cells, respectively (46). Further investigations have revealed that the invasion of SARS-CoV and HCoV-NL63 into host cells not only relies on the presence of ACE2 on host cell membrane as an entry receptor but also is modulated by other factors on host cell membranes such as HSPGs, which serve as attachment receptors (40, 45, 47).

At present, the mechanism of human CoV invasion into host cells is still not clear. Lang and Milewska described the possible mechanism of ACE2-mediated host cell entry for SARS-CoV and HCoV- NL63 virus (41, 45, 47). First, the virus docked and bound to host cells by the interaction between the spike protein on viral surface and heparan sulfate chains of HSPGs on host cell membrane. This action facilitated further binding of spike protein on viral surface to its entry receptor, ACE2, on host cell surface. Then, the binding of spike protein of the virus to ACE2 protein of host cell membrane triggered the recruitment of clathrin, followed by clathrin-mediated dynamin-dependent endocytosis of viral particles, which required actin cortex remodeling (39, 45, 47). Considering the 2019-nCoV has similar spike protein to SARS-CoV, and also uses ACE2 as its entry receptor to infect host cells, it is reasonable to presume that 2019-nCoV has the same invasive strategy for host cell entry as SARS-CoV and that HSPGs may also act as attachment receptors during the entry of 2019-nCoV into its host cells.

## Infection Control and Personal Protection

Patients infected by 2019-nCoV, similar to SARS cases, mostly present with non-specific symptoms such as fever, dry cough, and dyspnoea, or, in some cases, no evident symptoms, at the early phase of the disease (9, 16, 23–27, 48). Hence, it is a challenging task for health care professionals in the northern hemisphere to distinguish early 2019-nCoV infection from influenza and other respiratory viral infections in the seasons of winter and spring when respiratory diseases frequently break out (48). Hospital-related viral transmission, especially transmissions between patients and health-care workers, is frequently reported just before the outbreak of a highly infectious novel respiratory virus such as SARS-CoV and 2019-nCoV (8). Previous investigations have revealed that patients infected by a novel virus never identified before can easily transmit the pathogen to health personnel without enough personal protection; the latter getting infected will further become a source of spread and soon cause hospital-related viral transmission (8, 49–53). In fact, 386 of 1,755 patients (21.9%) and eight deaths were health-care workers during the SARS outbreak (49). As of February 11, 2020, a total of 3019 medical health workers have been infected by 2019-nCoV in China, among whom 1,716 cases were laboratory-confirmed CoVID-19, and five cases passed away, including an ophthalmologist named Wenliang Li, the whistleblower of 2019-nCoV infection in China (9, 16).

At present, the physicochemical properties of 2019-nCoV are not yet clear. Based on previous experience in SARS-CoV and MERS-CoV infection control, it is postulated that 2019-nCoV is sensitive to ultraviolet irradiation and heating. Sterilization can be achieved by heating at 56°C for 30 min and by lipid solvents including 75% ethanol, chlorine-containing disinfectant, peroxyacetic acid, and chloroform but not by chlorhexidine (50–52). Many ophthalmic instruments, i.e., probes for A-type and B-type ultrasound, ocular contact lenses such as the Goldmann three-mirrored lens and gonioscope, trial frames, slit-lamp microscope, direct ophthalmoscope, automatic perimeter, and fundus camera, are frequently used by direct or close contact with patients and may act as media for virus spread. A non-contact tonometer may create an aerosol when measuring intraocular pressure by punching air onto the cornea of patients; hence, it may also facilitate virus spread by aerosol transmission. Therefore, complete sterilization by 75% ethanol or hydrogen peroxide cleaning or immersion should be performed soon after each use of above the ophthalmic instruments (50–52). Complete sterilization using chlorine-containing disinfectant, peroxyacetic acid, and hydrogen peroxide is mandatory for clinics and operating rooms. Hand washing, preferably with the use of chlorhexidine alcoholic hand rub, after each ophthalmic examination or therapeutic procedure is highly recommended for the prevention of cross-infection.

Routine ophthalmic examinations such as slit-lamp examination and direct ophthalmoscopy are all performed by close contact, which means that the ophthalmologists can easily be exposed to the droplets and tears or ocular secretions from, or to the ophthalmic instruments contaminated by, patients with or suspected of having SARS, MERS, or CoVID-19. Hence, strict hand hygiene and proper personal protection equipment, including masks, gowns, gloves, and goggles, are highly recommended to avoid hospital-related viral transmission during ophthalmic practice (49–53).

When an ophthalmologist examines general ophthalmic outpatients, primary personal protection with disposable cap, surgical mask, and gown is recommended. When high-risk procedures are performed on these patients, for example, direct ophthalmoscopy, lacrimal irrigation and probing, intraocular pressure measurement with non-contact tonometry, ophthalmic laser therapy, and ophthalmic surgeries, N95 respirator, gloves, and goggles or face shield, are highly recommended (50). For patients with confirmed or suspect SARS, MERS, or CoVID-19, any ophthalmic consultation should be completed within the quarantine ward to avoid cross-infection. Personal protective equipment, including disposable caps, N95 respirator, goggles, face-shields, gloves, top and pants, and protective gowns, should be worn at all times (51, 53). Moreover, hand washing, preferably with the use of a chlorhexidine alcoholic hand rub, and gloves changed after each high-risk procedure are mandatory to prevent cross-infection. Ophthalmic personnel are also recommended not to touch their goggles, face shield, surgical/N95 mask, eye, head, and neck region before the handwashing procedure is completed (51, 53).

Non-urgent ophthalmic operations and interventions, for example, cataract operations, ophthalmic plastic surgeries, squint extraocular muscle surgeries, intravitreal anti-VEGF injection, retinal photocoagulation, and YAG: Nd laser capsulotomy should be delayed if possible (50–53). Ophthalmic emergencies such as acute angle-closure glaucoma and severe ocular injury should be operated upon immediately, but the operating theater should be regarded a high-risk area, and the use of proper personal protection equipment (i.e., disposable caps, N95 respirators, face shields, goggles, surgical gowns, and gloves) should be practiced strictly. When ophthalmic emergency surgeries are performed on patients with confirmed or suspected SARS, MERS, or CoVID-19, the recommended personal protection equipment are similar to those for ophthalmic consultation of these patients. To avoid aerosol transmission during tracheal intubation, local ophthalmic anesthesia is highly recommended rather than general anesthesia, and patients should wear N95 respirators during ophthalmic surgeries under local anesthesia (51, 53).

## Conclusion

The outbreak of the current 2019-nCoV infection has become a worldwide health threat. Although respiratory droplets and direct contact have been identified as the main routes of transmission for human CoVs, the role of the eye in transmitting human CoVs is still under discussion. Considering that the conjunctiva of the eye is directly exposed to infectious droplets and fomites during close contact with infected individuals and contaminated hands and that the mucosa of the ocular surface and the upper respiratory tract are connected by the nasolacrimal duct and share the same entry receptors for some respiratory viruses, it is reasonable to postulate three roles that the eye may play in human CoV infection. Firstly, it may be a target organ for human CoVs. Secondly, the conjunctiva may be a portal of entry for or a transporter of human CoVs to infect the respiratory tract. Thirdly, tears and conjunctival secretions may act as media that spread human CoVs. However, the eye is rarely involved in SARS-CoV, MERS-CoV, and 2019- nCoV infection; conjunctivitis has been reported in only five cases with CoVID-19 but never in SARS and MERS patients. This suggests that the eye is neither a preferred organ for human CoV infection nor a preferred gateway of entry that enables human CoVs to infect the respiratory tract.

Although it is quite rare, the possibility cannot be excluded that pathogens exposed to the eye might be transported to nasal and nasopharyngeal mucosa by constant tear rinsing through the lacrimal duct system and then cause respiratory tract infection, since mild to moderate symptomatic SARS can be developed in a cynomolgus macaques model by nasal and conjunctival SARS-CoV inoculation as well as by nasal and bronchial SARS-CoV inoculation (4, 54). Moreover, the extremely low positive rate of human CoV RNA tests by RT-PCR in tears and conjunctival secretions from patients with SARS and CoVID-19 may be related to the relatively low sensitivity of the current RT-PCR technique, later timing sample collection, and the activation of the host immune system and significant increases in lactoferrin and secretory IgA levels in tears and in circulating IgM and IgG levels in plasm. Hence, current negative RT-PCR results cannot exclude the possibility of the presence of SARS-CoV and 2019-nCoV in tears and conjunctival secretions. Considering that close doctor-patient contact is quite common in ophthalmic practice and is apt to transmit human CoVs via droplets and fomites, strict hand hygiene and proper personal protection are highly recommended for health care workers to avoid hospital-related viral transmission during ophthalmic practice.

## Author Contributions

CS and ZL performed the majority of the writing. CS, YW, and GL performed the literature review and data collection. ZL and CS revised the manuscript.

## Conflict of Interest

The authors declare that the research was conducted in the absence of any commercial or financial relationships that could be construed as a potential conflict of interest.
